# ATRX function beyond hippocampal CA1 is required for cognitive deficits in mouse models of intellectual disability

**DOI:** 10.1371/journal.pone.0347770

**Published:** 2026-04-28

**Authors:** Julia T. Brott, Nathalie G. Bérubé

**Affiliations:** 1 Department of Anatomy & Cell Biology, Western University, London, Ontario, Canada; 2 Department of Paediatrics, Western University, London, Ontario, Canada; 3 Department of Oncology, Western University, London, Ontario, Canada; 4 Division of Genetics & Development, Children’s Health Research Institute, London, Ontario, Canada; Zhengzhou University, CHINA

## Abstract

ATR-X syndrome, caused by mutations in the *ATRX* gene, leads to intellectual disability and neurodevelopmental deficits, with previous mouse models implicating forebrain ATRX loss in cognitive impairment. However, the region-specific requirements of neuronal ATRX for cognitive function remain unclear. Here, we generated conditional knockout mice with predominant deletion of ATRX in hippocampal CA1 pyramidal neurons in both pure C57Bl/6J and hybrid C57Bl/6J + 129S2/Sv genetic backgrounds. Immunofluorescence confirmed efficient ATRX loss in CA1 neurons, with mosaic expression throughout other forebrain structures. Behavioral analyses revealed that T29-1 CaMKIIα-Cre ATRX knockout mice exhibited significant hypoactivity and increased anxiety traits, particularly in the open field, but retained normal hippocampal-dependent contextual fear memory and spatial learning and memory. In contrast, we confirmed that mice with robust forebrain-wide ATRX ablation in excitatory neurons (R1ag#5 CaMKIIα-Cre-mediated) displayed deficits in these cognitive domains. Our findings demonstrate that ATRX-related intellectual disability requires disruption of broader hippocampal or forebrain circuits to elicit cognitive impairments in learning and memory.

## Introduction

Intellectual disability (ID) is a major neurodevelopmental challenge affecting millions worldwide, with chromatin remodeling factors increasingly recognized as key regulators of brain development and cognitive function. α-thalassemia X-linked intellectual disability (ATR-X) syndrome is a congenital neurodevelopmental disorder characterized by moderate-to-severe intellectual disability in male paediatric patients with hypomorphic mutations in the *ATRX* gene [1]. ATRX is ubiquitously expressed throughout the human brain, present in progenitors and post-mitotic cell types, including neurons and glia, with high expression observed in the cerebral cortex and hippocampus [2,3]. The ATRX protein is an ATP-dependent chromatin remodeler that plays critical roles in histone variant deposition, regulation of DNA replication and repair, maintenance of telomere integrity and transcriptional regulation [4–10].

The critical role of ATRX in brain development is underscored by the embryonic lethality observed following global deletion and the severe neuroanatomical and behavioral abnormalities resulting from forebrain- or CNS-specific conditional knockout models [11–16]. Previously, conditional knockout of ATRX in postnatal forebrain excitatory neurons resulted in learning and memory impairments, as well as alterations in the hippocampal cornu ammonis (CA1) region [13,17]. This included increased volume of CA1 layers: stratum radiatum and stratum lacunosum moleculare, ultrastructural synaptic changes, and reduced long-term potentiation (LTP) when stimulated directly through the temporoammonic pathway, or indirectly through the Schaffer collaterals [13,17]. The CA1 pyramidal neurons are central to hippocampal-dependent memory formation and retrieval, and alterations in their synaptic plasticity have been directly linked to cognitive performance [18–20]. While forebrain-wide ATRX loss produces cognitive deficits, it is unclear whether disruption of ATRX in individual hippocampal subregions, such as the CA1, is sufficient to impair learning and memory.

In the present study we generated and characterized mice with a predominant conditional deletion of ATRX in hippocampal CA1 pyramidal neurons, using the αCaMKII-Cre T29-1 line. As cognitive performance in behavioural tasks has been shown to vary between common inbred mouse strains, we conducted these experiments using both a pure C57Bl/6J and hybrid C57Bl/6J + 129S2/Sv genetic background [21]. Previous spatial learning and long-term memory tests have reported that 129/Sv mice perform comparably to C57BL/6J mice, although sub-strain differences in the 129 family have been shown to affect hippocampal-dependent behaviour [22–24]. We hypothesized that T29-1 mediated ATRX loss would be sufficient to disrupt hippocampal-dependent cognitive function. Through a comprehensive battery of behavioral assays, we evaluated the impact of T29-1 conditional ATRX deletion on locomotion, anxiety, and memory, providing new insights into the regional requirements for ATRX in the hippocampus and the etiology of cognitive deficits in ATR-X syndrome.

## Materials and methods

### Mouse models

All procedures involving animals were conducted in accordance with the regulations of the Animals for Research Act of the province of Ontario and approved by the University of Western Ontario Animal Care and Use Committee (2021−049). Mice harbouring a predominant CA1 excitatory neuron deletion of *Atrx* were generated by crossing the previously described Cre-sensitive *Atrx*^loxP^ mice with CaMKIIα-Cre T29-1 transgenic mice purchased from The Jackson Laboratory [B6.Cg-Tg(Camk2a-cre)T29-1Stl/J; Strain #:005359; RRID:IMSR_JAX:005359] [11,25]. The progeny include mice with loss of ATRX expression in the pyramidal cells of hippocampal CA1 beginning at post-natal day 20 [25]. Pure C57Bl/6J background *Atrx*^+/Y^;Cre^+/-^ (Ctrl^B6^) and *Atrx*^f/Y^;Cre^+/-^ (cKO^B6^) male mice were generated by crossing T29-1 Cre^+/-^ males with heterozygous *Atrx*^f/+^ female C57Bl/6J mice [RRID:IMSR_JAX:000664]. Hybrid C57Bl/6J + 129S2/Sv *Atrx*^+/Y^;Cre^+/-^ (Ctrl^Hybrid^) and *Atrx*^f/Y^;Cre^+/-^ (cKO^Hybrid^) male mice were generated by crossing T29-1 Cre^+/-^ males with heterozygous *Atrx*^f/+^ female 129S2/Sv mice [RRID:IMSR_CRL:287]. Mice harbouring a forebrain excitatory neuron deletion of *Atrx* were generated by crossing *Atrx*^loxP^ 129S2/Sv mice with CaMKIIα-Cre R1ag#5 transgenic mice purchased from The Jackson Laboratory [B6.Cg-Tg(Camk2a-cre)2Szi/J; Strain #: 027310; RRID:IMSR_JAX:027310] [11,26]. Progeny from this cross include mice lacking ATRX expression in excitatory neurons of the forebrain beginning at post-natal day 5 [26]. Post-weaning, mice were group-housed (2−4 per cage) in standard shoebox cages with bedding and nesting materials, located in ventilated racks. Mice were exposed to a 12-hour-light/12-hour-dark cycle with water and chow ad libitum. Ear notch biopsies were taken at P10 for animal identification by PCR genotyping as previously described using the primers in S1 Table [11]. Mice were checked daily for any signs of distress including, but not limited to, wounds/ruffled fur, changes in appearance, abnormal behaviors, reduced mobility, and altered body posture. However, no animals died before meeting their experimental endpoint. All mice reached experimental endpoint for immunofluorescence (3 months, N = 6) or for behavioural assessments (4−6 months, N = 93) and were euthanized by CO_2_ inhalation with secondary cervical dislocation, as described in the animal use protocol (2021−049). All research staff received the following training from Animal Care and Veterinary Services at Western University: Animal Ethics and Regulation, Mouse Handling & Care, Rodent Gas Anesthesia, and Cervical Dislocation with Anesthesia.

### Immunofluorescence of brain sections

Mice were perfused with ice cold phosphate buffered saline (PBS), followed by ice cold 4% PFA diluted in PBS. The brain was extracted and placed overnight in fresh 4% PFA/PBS. The following day, brains were washed three times in PBS and stored in 30% sucrose in PBS until fully saturated. Brains were embedded in Cryomatrix™ [Epredia™; 67-690-06] and frozen in an ‘ethanol-dry-ice slurry’ and stored at −80°C. Serial coronal 10μM cryosections of the caudal cortical/hippocampal region were obtained using a Leica cryostat [CM3050 S; RRID:SCR_016844]. Sections were rehydrated in PBS and underwent antigen retrieval by placing slides in a boiling 10Mm sodium citrate solution pH 6.0 for 15 minutes. Sections were then washed with PBS and processed according to the M.O.M.® (Mouse on Mouse) Immunodetection Kit [VECTBMK2202] manufacturers protocol, with the notable exception that primary antibodies were incubated on sections overnight at 4°C. Primary antibodies used were polyclonal anti-rabbit ATRX [1:200; Santa Cruz; sc-15408; RRID:AB_2061023] and monoclonal anti-mouse NeuN [1:200; Millipore Sigma; MAB377; RRID:AB_2298772]. Secondary antibodies were used at a dilution of 1:1000 as appropriate: Streptavidin, Alexa Fluor™ 647 Conjugate [ThermoFisher Scientific; S32357] and Goat anti-Mouse Alexa Fluor™ 488 [ThermoFisher Scientific; A-11017; RRID:AB_3668622]. Sections were treated with DAPI stain [Millipore Sigma; D9542] for 5 minutes at room temperature, washed three times with PBS, and mounted using PermaFluor™ Mounting Medium [Epredia™; TA-030-FM]. Immunostained cryosections were imaged on a Leica DM6000 B inverted fluorescence microscope equipped with a Hamamatsu ORCA-ER-1394 digital camera using Volocity acquisition software [RRID:SCR_002668]. Image processing and adjustments were carried out using Volocity [Demo Version 6.0.1] and Adobe Photoshop [Version 20.0.4; RRID:SCR_014199].

### Behaviour testing

Behaviour experiments were performed with mice between 4- to 6-months of age, in order of least demanding (open field and elevated plus maze) to most demanding (contextual fear conditioning and Morris water maze). ARRIVE guidelines were followed: animal groups were randomized, experimenters were blind to the genotypes, software-based analysis was used to score performance in all tasks. All tests were conducted between 9:00AM and 4:00PM.

#### Open field.

Mice were placed in a 20 cm x 20 cm open arena with 30 cm high walls [VersaMax Legacy Open Field] and left to explore for 2-hours. Animals were allowed to habituate to the room for 30-minutes prior to testing while in their home cages. Distance travelled, as well as distance and time spent in center were automatically recorded every 5-minutes [AccuScan Instrument].

#### Elevated plus maze.

Animals were placed in the center of an elevated plus maze apparatus [Med Associate Inc] composed of three zones: two open arms, two closed arms, and a center square. Mice were allowed to freely move around the apparatus for a period of 5-minutes. ANY-maze video tracking software [RRID:SCR_014289] was used to determine which zone they were in as indicated by the center on their bodies.

#### Contextual fear memory.

Fear memory was tested using a 20 cm x 10 cm clear acrylic enclosure with a metal grid floor equipped to generate 180V foot-shocks. To distinguish the apparatus one wall had stripes drawn on it while the wall opposite had a star. On the first day, mice were conditioned to the apparatus over a period of 3-minutes, with a 2-second foot-shock being administered at 2.5-minutes. On day 2, mice were returned to the chamber and the time spent immobile was measured over a period of 6-minutes. Videos were recorded and analyzed using the ANY-maze video tracking software [RRID:SCR_014289]. Time spent immobile was determined as the amount of time in which an animal was immobile for a minimum period of 0.5 seconds with the sensitivity in ANY-maze set to 95%.

#### Morris water maze.

The Morris water maze task was conducted as previously described with minor modifications [13,14,27]. Briefly, animals underwent four 90-second training trials, separated by a 15-minute interval, each day for four consecutive days to learn to find a submerged (1.5 cm below water surface) platform. When mice were unsuccessful at finding the platform during the 90-second trial period, they were guided to the platform. On the fifth and twelfth days the platform was removed from the pool and mice were placed in the pool for a 1-minute probe trial. ANY-maze video tracking software [RRID:SCR_014289] was used to measure distance, swim speed, latency, and time spent in each quadrant of the maze.

### Statistical analysis

All statistical analysis, except for the Dirichlet uniformity test, was conducted using GraphPad Prism 9 [RRID:SCR_002798]. Comparison of Ctrl and cKO mice in the open field, contextual fear memory, and elevated plus maze was determined using a two-way ANOVA with a Bonferroni-Sidak for multiple comparisons for analysis over time and an unpaired t-test for direct comparison of Ctrl versus cKO mice. A two-way ANOVA with a Bonferroni-Sidak test for multiple comparisons was conducted for the analysis of distance, mean speed, and latency during the training phases of the Morris water maze, while a two-way ANOVA with a Dunnett test for multiple comparisons was done for time spent in each quadrant during the probe trials. The Dirichlet uniformity test was conducted as previously described in Maugard and Doux, 2018 using the online python platform Binder [28]. A summary of all statistical analyses can be found in S2 Table.

## Results

### Generating mice with deletion of ATRX in CA1 pyramidal hippocampal neurons

**Fig 1:**
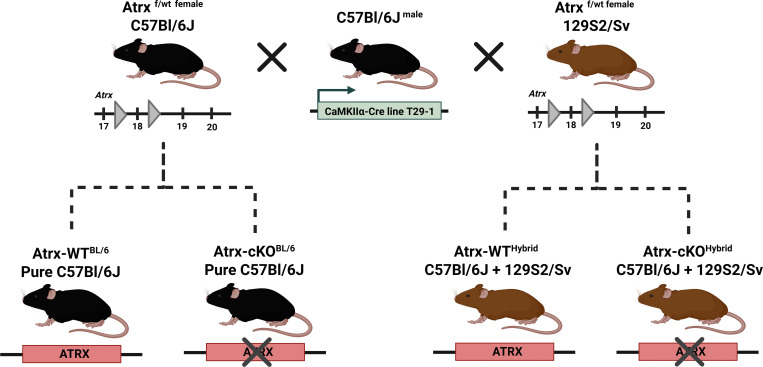
Generation of T29-1 CaMKIIα-Cre ATRX knockout mice. Schematic of the breeding strategy used to generate mice with a predominant conditional deletion of ATRX in hippocampal CA1 excitatory neurons. Male B6.Cg-Tg(Camk2a-cre)T29-1Stl/J mice were crossed with female mice carrying loxP-flanked (floxed) exon 18 of the *Atrx* gene to produce offspring on a pure C57Bl/6J or hybrid C57Bl/6J + 129S2/Sv background. Cre-mediated recombination results in conditional loss of ATRX protein primarily in CA1 pyramidal neurons beginning around postnatal day 20. Reprinted from Science Suite Inc. dba BioRender (“BioRender”) under a CC BY license, with permission from BioRender, original copyright 2026. Created in BioRender. Brott, **J.** (2026) https://BioRender.com/epud9qw.

**Fig 2:**
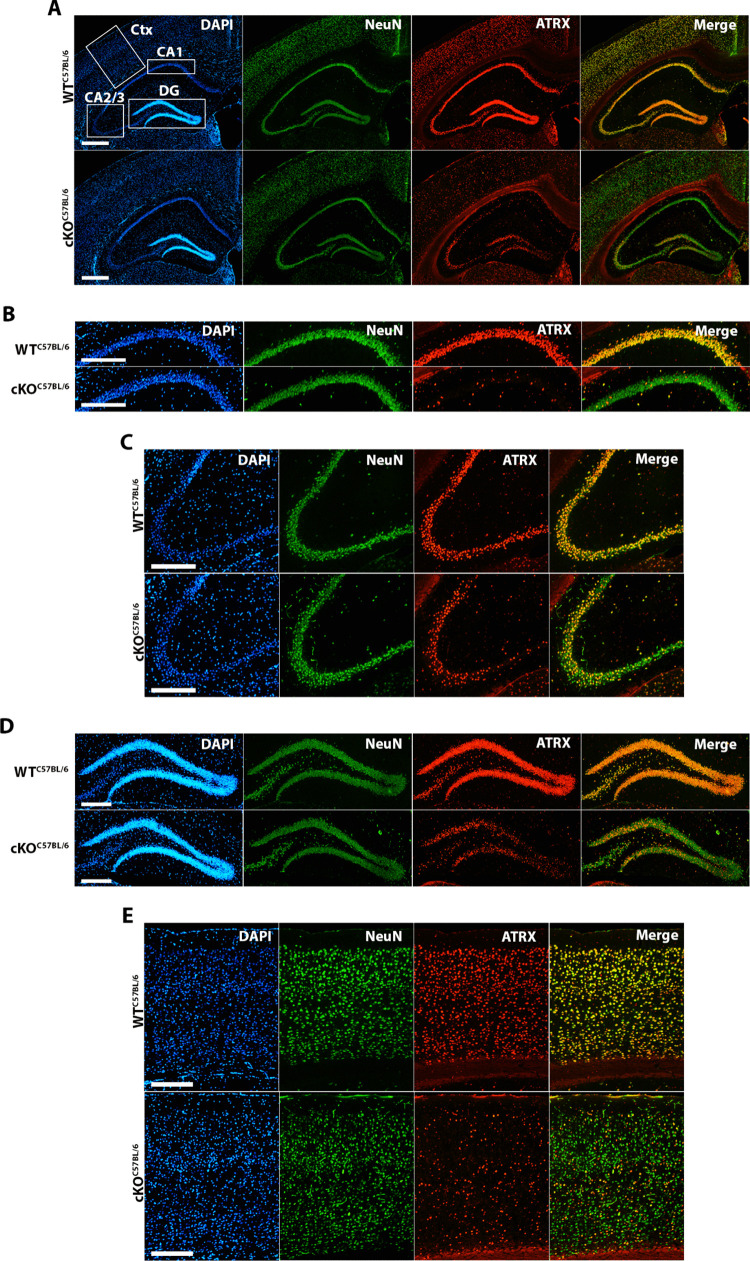
Overview of T29-1 CaMKIIα-Cre-mediated ATRX deletion in a C57Bl/6J background. **(A)** Representative immunofluorescence images of coronal brain sections from 3-month-old control and ATRX cKO C57Bl/6J mice. Sections were stained for ATRX (red), the neuronal marker NeuN (green), and the nuclear marker DAPI (blue). Images were captured at 5X magnification; scale bar represents 500μm. Higher magnification images of ATRX and NeuN expression in (B) the hippocampal cornu ammonis 1 (CA1), (C) hippocampal CA2/3, (D) dentate gyrus (DG), and (E) the cortex (Ctx). Scale bars represent 250μm.

Our goal was to generate mice that lack *Atrx* expression in hippocampal CA1 pyramidal excitatory neurons using the purported sub-region specific αCaMKII-Cre driver line T29-1 [25]. The alpha subunit of the calcium/calmodulin-dependent protein kinase II (αCaMKII) is expressed in excitatory forebrain neurons, while the unique transgene integration site in the T29-1-line is suggested to result in Cre/loxP recombination during the third post-natal week that is primarily confined to the hippocampal CA1 region [25,29]. Male C57Bl/6J mice carrying the T29-1 transgene were mated with either a C57Bl/6J or 129S2/Sv female heterozygous for the *Atrx* floxed allele to generate experimental mice (Fig 1). Loss of ATRX expression was confirmed at 3-months by immunofluorescence staining of coronal brain sections, in both the pure C57Bl/6J and hybrid C57Bl/6J + 129S2/Sv backgrounds (Fig 2A) (S1 Fig). The results show that ATRX protein is robustly lost in NeuN + CA1 neurons throughout both the dorsal and ventral hippocampus (Fig 2B) (S1-S2 Figs). A partial loss of immunoreactivity was observed in NeuN+ neurons in hippocampal CA2/3, the dentate gyrus and the cerebral cortex (Fig 2C-E) (S1 Fig). This highlights that Cre expression mediated by the T29-1 CaMKIIα transgene results in mosaic expression of ATRX throughout the forebrain, with predominant knockout occurring in the CA1 pyramidal cell layer, consistent with other reports [30–32]. A similar pattern of ATRX inactivation is observed in C57Bl/6J background mice compared to the hybrid C57Bl/6J + 129S2/Sv background mice, suggesting that there is no strain effect on the efficiency of Cre/loxP recombination.

### Strain independent hypoactivity observed in T29-1 CaMKIIα-Cre ATRX cKO mice

**Fig 3:**
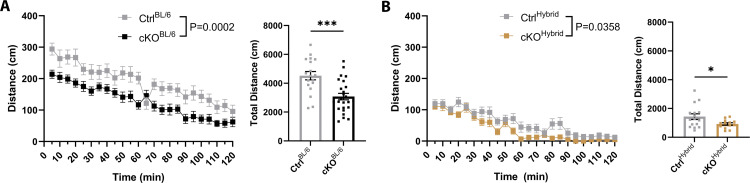
T29-1 CaMKIIα-Cre-mediated ATRX deletion induces hypoactivity in the open field. **(A)** Distance traveled by 4–6-month-old C57Bl/6J male control (n = 18) and cKO (n = 25) mice. **(B)** Distance traveled by 4–6-month-old hybrid male control (n = 16) and cKO (n = 13) mice. Statistical significance across time was determined using two-way ANOVA with Bonferroni-Šídák correction for multiple comparisons; total distance traveled was compared using unpaired t-tests. *p < 0.05, *** p < 0.0005.

Exploratory behaviour and locomotor activity were evaluated using the open field test, where mice were placed into an empty square arena and allowed to explore freely for two hours. This was conducted to establish baseline performance and assess activity levels, as many anxiety, and spatial learning and memory tests rely on body activity and movement [33,34]. Consistent with established strain differences, control hybrid C57Bl/6J + 129S2/Sv (Ctrl^Hybrid^) mice are significantly less active than control C57Bl/6J (Ctrl^BL/6^) mice (p < 0.0001, F = 2.455) (S3 Fig), with Ctrl^BL/6^ mice traveling approximately three times the distance of their hybrid counterparts (Ctrl^BL/6^ mean distance = 4524 cm; Ctrl^Hybrid^ mean distance = 1434 cm). Importantly, T29-1 cKO mice exhibited a significant reduction in total distance traveled compared to their respective controls, regardless of genetic background (BL/6: p = 0.0002, F = 1.180; Hybrid: p = 0.0266, F = 6.471) (Fig 3A-B). This robust hypoactivity phenotype was not due to altered habituation as both genotypes exhibit the same decrease in exploratory behaviour over time as indicated by the non-significant interaction term (BL/6 p = 0.2936; Hybrid p = 0.4123). This indicates that conditional loss of ATRX driven by the T29-1 transgene leads to decreased exploratory locomotion, independent of background strain.

### ATRX cKO^BL/6^ mice display increased anxiety in open field test

**Fig 4:**
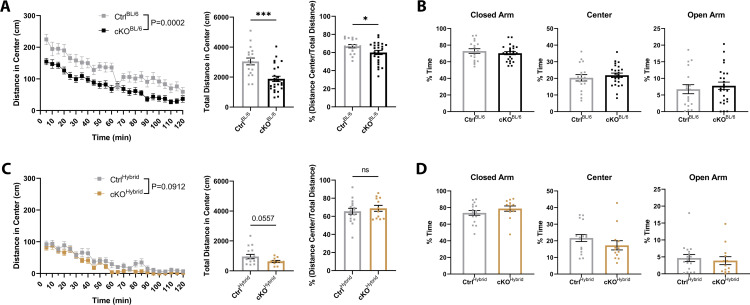
T29-1 CaMKIIα-Cre-mediated ATRX deletion increases anxiety-like behavior in cKO^BL/6^ mice. **(A)** Distance travelled in the center of the open field and (B) percentage of time spent in the corresponding area of the elevated plus maze for C57Bl/6J control (n = 18) and cKO (n = 25) male mice. **(C)** Distance travelled in the center of the open field and (D) percentage of time spent in the corresponding area of the elevated plus maze for hybrid control (n = 17) and cKO (n = 13) male mice. Statistical significance across time was determined using two-way ANOVA with Bonferroni-Šídák correction for multiple comparisons; unpaired t-tests were used to compare total center distance in the open field and time spent in each EPM area. * p < 0.05, *** p < 0.0005.

Previous studies have shown that loss of ATRX can affect anxiety-related behaviours, and since decreased distance traveled in the open field test can be a result of increased anxiety-like behaviour we chose to test anxiety in the mice by analysing time in center of the open field arena [13,14,35]. Time in center was significantly decreased for cKO^BL/6^ but not cKO^Hybrid^ mice (BL/6: p = 0.0445, F = 1.186; Hybrid: p = 0.3341, F = 6.500) (S3 Fig). Given that mutant mice exhibited reduced locomotor activity in the open field, we further assessed anxiety-like behaviour using locomotion-normalized measures. Distance traveled in the center was measured and analyzed as a percentage of total distance travelled. Both cKO^BL/6^ and cKO^Hybrid^ mice exhibited reduced total center distance relative to controls, but statistical significance was reached only in the cKO^BL/6^ group (BL/6: p = 0.0002, F = 1.188; Hybrid: p = 0.0557, F = 6.808) (Fig 4A, 4C). When normalized to total distance travelled in the open field, only cKO^BL/6^ demonstrate reduced center distance relative to controls (BL/6: p = 0.0364, F = 2.179; Hybrid: p = 0.4972, F = 1.316). To further assess anxiety independently of hypoactivity, we employed the elevated plus maze EPM, which introduces an anxiogenic challenge by exposing mice to open, elevated arms [36]. To control for the observed hypoactivity of T29-1 cKO mice the total distance traveled and mean speed in the EPM was measured. Consistent with the total distance traveled in the open field test, Ctrl^BL/6^ mice move significantly more than Ctrl^Hybrid^ mice in the EPM (Ctrl^BL/6^ mean distance = 11.60m; Ctrl^BL/6^ mean distance = 6.637m, p < 0.0001, F = 2.697) (S3 Fig). No difference was observed between T29-1 cKO mice and their corresponding controls, in either strain background for total distance traveled or mean speed (BL/6: total distance p = 0.1555, mean speed p = 0.1626; Hybrid: total distance p = 0.0580, mean speed p = 0.0652) (S3 Fig). No significant differences were identified in either strain background when comparing percent of time spent in the closed arm, center, or open arm, of the EPM (BL/6: closed p = 0.4344, center p = 0.4875, open p = 0.5596; Hybrid: closed p = 0.2356, center p = 0.2094, open p = 0.6249) (Fig 4B, 4D). This pattern indicates that the anxiety phenotype is context-dependent and may reflect increased anxiety traits rather than heightened state anxiety.

### Intact memory in T29-1 CaMKIIα-Cre ATRX cKO mice

**Fig 5:**
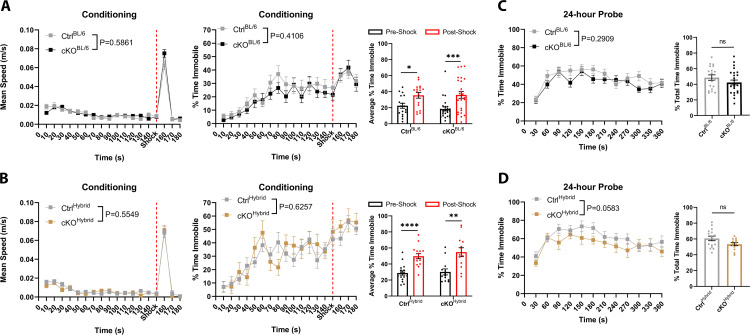
T29-1 CaMKIIα-Cre-mediated ATRX deletion does not impair contextual fear memory. **(A)** Contextual fear conditioning in 4–6-month-old male control (n = 18) and cKO (n = 25) mice in a pure C57Bl/6J background, and (B) in control (n = 16) and cKO (n = 13) mice in a hybrid C57Bl/6J + 129S2/Sv background. Mice were placed in the conditioning chamber for 180 seconds, with a 2-second, 180V foot shock delivered at 150 seconds (indicated by red dotted line). **(C, D)** Contextual fear memory was assessed 24 hours later by measuring the percentage of time spent immobile (freezing; defined as immobility >0.5 seconds) in the same context for both pure C57Bl/6J (C) and hybrid (D) backgrounds. Statistical significance for changes over time was determined using two-way ANOVA with Bonferroni-Šídák correction for multiple comparisons; unpaired t-tests were used for comparisons of percent total time immobile between groups. * p < 0.05, *** p < 0.0005, **** p < 0.00005.

As mutations in *ATRX* result in intellectual disability in patients, a common phenotype observed in mouse models are cognitive deficits, assessed using both spatial learning and fear memory paradigms [13,14,16,37,38]. To focus on hippocampal-dependent memory, we first tested contextual fear memory by placing the mice into a rectangular box with two distinct visual cues, on opposing walls, and a metal grate floor, for a period of 180-seconds [39,40]. At the 150-second mark, a 2-second 180V foot shock was administered through the metal grate floor. To ensure mice were properly conditioned and responded to the foot shock, both mean speed and percent time immobile was measured. We observed that Ctrl and cKO mice, in both strains, respond to the foot shock with a brief period of rapid movement followed, or darting, demonstrating shock perception by the animals (Fig 5A-B) [41]. To determine if fear acquisition was successful, the percentage of time immobile (freezing behaviour > 0.5s) was compared pre- and post-shock. Both Ctrl and cKO animals spent significantly more time immobile post-shock, suggesting an increase in freezing behaviour (Ctrl^BL/6^: p = 0.0102 cKO^BL/6^: p = 0.0008; Ctrl^Hybrid^: p=<0.0001; cKO^Hybrid^: p = 0.0012) (Fig 5A-B). Fear memory was measured 24-hours post-conditioning by placing animals back into the apparatus and measuring the percent of time spent immobile over a period of 5-minutes. We found that both cKO^BL/6^ and cKO^Hybrid^ mice spend the same percentage of time immobile as their control counterparts (BL/6: p = 0.2908; Hybrid: p = 0.0583) (Fig 5C-D). Additionally, we noted that Ctrl^Hybrid^ mice freeze significantly more than Ctrl^BL/6^ mice during the 24-hour probe (p = 0.0126, F = 1.660) (S3 Fig).

**Fig 6:**
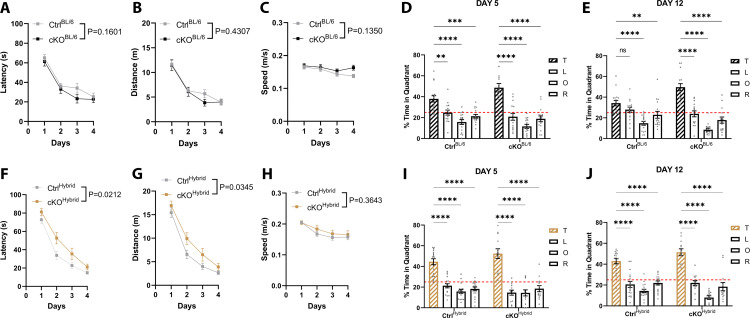
T29-1 CaMKIIα-Cre-mediated ATRX deletion does not affect spatial learning and memory in the Morris water maze. **(A–C)** Spatial learning performance of C57Bl/6J male control (n = 16) and cKO (n = 15) mice over four consecutive training days. **(A)** Latency to locate the submerged platform, (B) distance traveled to reach the platform, and (C) mean swimming speed were recorded to assess learning and motor function. **(F–H)** Corresponding spatial learning measures for hybrid control (n = 17) and cKO (n = 13) mice: (F) latency, (G) distance traveled, and (H) mean swimming speed. (D, E, I, **J)** Spatial memory was evaluated in probe trials following platform removal. The percentage of time spent in each of the four quadrants was recorded for C57BL/6J mice (D, 24-hour probe; E, day 12 probe) and hybrid background mice (I, 24-hour probe; J, day 12 probe). Statistical analyses were performed using two-way ANOVA with Bonferroni-Šídák correction for training data (A–C, F–H) and Dunnett’s multiple comparisons test for probe trials. ** p < 0.005, *** p < 0.0005, **** p < 0.00005.

Spatial learning and memory were tested using the Morris water maze (MWM) paradigm. Mice were placed in a circular pool and trained over four consecutive days to locate a submerged escape platform, using four distinct spatial cues positioned around the testing room. Across the training period, all mouse groups demonstrated robust learning, as evidenced by progressive reductions in both latency and distance to reach the platform (Fig 6A-B, 6F-G). Swimming speed remained consistent between cKO and control mice for both genetic backgrounds throughout the training sessions, indicating that motor performance or motivation did not confound the learning assessment (Fig 6C, 6H). To probe spatial memory the platform was removed and the time spent in each quadrant of the tub over a period of 60-seconds was measured. On day 5, all groups had intact short-term spatial memory as they spent significantly more time in the target quadrant which previously contained the submerged platform, compared to the other three quadrants (Ctrl^BL/6^: T vs. L p = 0.0026, T vs. O p < 0.0001, T vs. R p = 0.0001; cKO^BL/6^: T vs. L p < 0.0001, T vs. O p < 0.0001, T vs. R p < 0.0001; Ctrl^Hybrid^: T vs. L p < 0.0001, T vs. O p < 0.0001, T vs. R p < 0.0001; cKO^Hybrid^: T vs. L p < 0.0001, T vs. O p < 0.0001, T vs. R p < 0.0001) (Fig 6D, 6I). Long-term spatial memory, evaluated 8 days post-training (day 12), was similarly preserved, with all groups again exhibiting a clear preference for the target quadrant (Ctrl^BL/6^: T vs. L p = 0.2261, T vs. O p < 0.0001, T vs. R p = 0.0091; cKO^BL/6^: T vs. L p < 0.0001, T vs. O p < 0.0001, T vs. R p < 0.0001; Ctrl^Hybrid^: T vs. L p < 0.0001, T vs. O p < 0.0001, T vs. R p < 0.0001; cKO^Hybrid^: T vs. L p < 0.0001, T vs. O p < 0.0001, T vs. R p < 0.0001) (Fig 6E, 6J). Collectively, these results demonstrate that conditional loss of ATRX mediated by the T29-1 transgene does not impair spatial learning or memory in either C57Bl/6J or hybrid C57Bl/6J + 129S2/Sv backgrounds. This finding contrasts with the pronounced cognitive deficits observed in broader forebrain ATRX knockout models, underscoring that ATRX function outside the CA1 region is likely required to disrupt hippocampal-dependent cognitive processes [13, 14].

### Generation of mice with robust forebrain excitatory neuron-specific deletion of ATRX

Previously our lab generated a mouse line harbouring a whole forebrain deletion of ATRX in excitatory neurons using the αCaMKII-Cre drive line L7Ag13 that is no longer available [13,26]. To enable a direct comparison between the presented T29-1 model and the previously described whole forebrain model, we used the closely related αCaMKII-Cre driver line R1ag#5 [26]. The transgene integration site in both the L7Ag13 and R1ag#5 lines results in Cre/loxP recombination at high levels throughout all forebrain structures starting at post-natal day 5 [26]. Loss of ATRX expression was confirmed at 3-months by immunofluorescence staining of the hippocampus and caudal cortical region in coronal brain sections (S4 Fig). The results show that ATRX protein is absent in NeuN+ neurons throughout all regions of the hippocampus proper (CA1-CA3), as well as the dentate gyrus and cerebral cortex.

### ATRX cKO^R1ag#5^ mice exhibit anxiolytic behaviour and memory deficits

**Fig 7:**
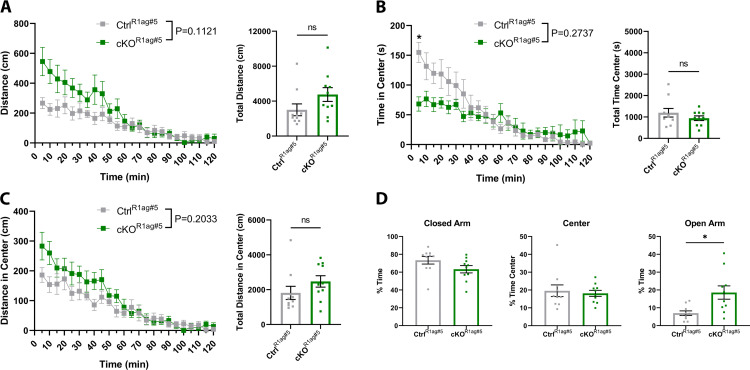
R1ag#5 CaMKIIα-Cre-mediated ATRX deletion reduces anxiety-like behaviour in male mice. **(A)** Distance traveled, (B) time in center, and (C) distance travelled in the center by 4–6-month-old hybrid male control (n = 10) and cKO (n = 10) R1Ag#5-Camk2a-Cre mice. **(D)** Percentage of time spent in each arm/zone of the elevated plus maze for the same cohorts. Statistical comparisons across timepoints were performed using two-way ANOVA with Bonferroni-Šídák correction for multiple comparisons. Group differences in total distance, center metrics in the open field, and time distribution in the elevated plus maze were assessed by unpaired t-tests. * p < 0.005.

We proceeded to validate the previously reported behavioural phenotypes observed in the L7Ag13 mouse model in the comparable R1ag#5 strain, starting with the open field test. Consistent with previous findings, cKO^R1ag#5^ mice do not show a significant difference in total distance traveled over the two-hour period (p = 0.1121, F = 1.346) (Fig 7A) [13]. However, unlike the L7Ag13 model, cKO^R1ag#5^ mice do not spend significantly more time or travel a significantly longer distance in the center of the open field apparatus (time in center p = 0.2737, F = 3.385, distance in center p = 0.2033, F = 1.324) (Fig 7B-C). To further assess anxiety-related behaviour, we employed the elevated plus maze EPM to simulate an anxiogenic challenge [36]. cKO^R1ag#5^ mice spend significantly more time in the open arms of the EPM (p = 0.0138, F = 8.537), confirming that the anxiolytic phenotype previously observed in L7Ag13 mice is maintained in the R1ag#5 model (Fig 7D).

**Fig 8:**
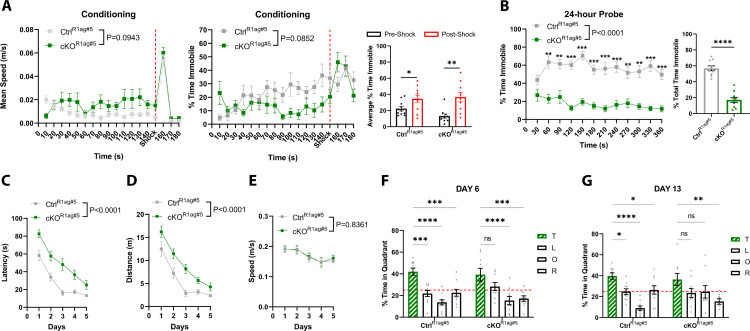
R1ag#5 CaMKIIα-Cre-mediated ATRX deletion impairs memory in male mice. **(A)** Contextual fear conditioning in 4–6-month-old male control (n = 10) and cKO (n = 10) R1Ag#5-Camk2a-Cre mice on a hybrid genetic background. Mice were placed in the conditioning chamber for 180 seconds; a 2-second, 180V foot shock was delivered at 150 seconds (denoted by the red dotted line). **(B)** Contextual fear memory was evaluated 24 hours later by quantifying the percentage of time spent freezing (immobility >0.5 seconds) in the same context. **(C–E)** Spatial learning in the Morris Water Maze over four consecutive training days: (C) latency to locate the submerged platform, (D) distance traveled to reach the platform, and (E) mean swimming speed were recorded. **(F, G)** Spatial memory was assessed in probe trials following platform removal by measuring the percentage of time spent in each quadrant (F: 24-hour probe trial; G: day 13 probe trial). Changes over time in conditioning and training were analyzed by two-way ANOVA with Bonferroni-Šídák correction for multiple comparisons. Percent total freezing between genotypes was compared using unpaired t-tests. Probe trials were analyzed by two-way ANOVA with Dunnett’s multiple comparisons test. *p < 0.05, **p < 0.005, ***p < 0.0005, ****p < 0.00005.

We next examined hippocampal-dependent memory in the mice [39,40]. In the contextual fear memory paradigm, both Ctrl^R1ag#5^ and cKO^R1ag#5^ mice respond to the foot shock with an initial period of rapid movement, or darting, followed by increased freezing behaviour (Ctrl^R1ag#5^: p = 0.0420, F = 2.841; cKO^R1ag#5^: p = 0.0013, F = 4.460) (Fig 8A). However, when fear memory was assessed 24-hours post-shock, cKO^R1ag#5^ mice exhibited significantly reduced freezing behaviour compared to controls, both over time (genotype interaction p < 0.0001) and in total immobility duration (p < 0.0001, F = 1.088) (Fig 8B). Spatial learning and memory were tested using the MWM paradigm. During the learning trials cKO^R1ag#5^ mice demonstrated significantly increased latency to locate the platform on training days 1–4 (genotype interaction p < 0.0001), but by day 5 their performance matched that of controls (Fig 8C). This learning deficit was further evident from the significantly longer distances travelled on training days 3–4 to reach the platform (genotype interaction p < 0.0001) (Fig 8D). Importantly, swimming speed remained consistent between genotypes throughout the five training days, indicating that motor performance or motivation did not account for the observed deficits (Fig 8E). Short-term spatial memory assessed on day 6 (24-hour probe), remained intact in the cKO^R1ag#5^ mice as they spent significantly more time in the target quadrant compared to two of the other three quadrants (Ctrl^R1ag#5^: T vs. L p = 0.0003, T vs. O p < 0.0001, T vs. R p = 0.0004; cKO^R1ag#5^: T vs. L p = 0.0901, T vs. O p < 0.0001, T vs. R p = 0.0002) (Fig 8F). Although the comparison with the left “L” quadrant was not significant, the overall interaction term was not significant (p = 0.3683) and the Dirichlet uniformity test showed that the time spent in the target quadrant is significantly higher leading to a non-uniform distribution (p = 0.0078) (S5 Fig). In contrast, long-term spatial memory evaluated 8 days post-training (day 13) was impaired. cKO^R1ag#5^ mice failed to show preferential exploration of the target quadrant compared controls (interaction term p = 0.0113) and the Dirichlet uniformity test showed mice displayed a uniform distribution between quadrants (p = 0.062) (Fig 8G) (S5 Fig). Collectively, these findings demonstrate that cKO^R1ag#5^ mice exhibit, anxiolytic behaviour, fear memory deficits, impaired spatial learning, and compromised long-term spatial memory. This aligns with the previously described L7Ag13 mice, as well as other characterized ATRX mouse models [13,14,16,37,38].

## Discussion

In this study, we aimed to resolve whether the loss of ATRX expression restricted to pyramidal excitatory neurons in hippocampal CA1 is sufficient to produce cognitive deficits in two commonly used mouse strains. We find that the T29-1 CaMKIIα-Cre transgene results in prominent deletion of ATRX in CA1 pyramidal neurons, with mosaic expression of ATRX in other forebrain structures, including the cerebral cortex, hippocampal CA2−3 and dentate gyrus. T29-1 CaMKIIα-Cre-driven deletion of ATRX does not impair contextual fear memory or spatial learning and memory as T29-1 cKO mice from both genetic backgrounds perform comparably to controls. In contrast, we validated that mice with ATRX ablation robustly throughout the forebrain display decreased anxiety, significant impairments in contextual fear memory, and deficits in spatial learning and long-term memory. Notably, we also found that loss of ATRX mediated by the T29-1 CaMKIIα-Cre transgene produces a strain-independent hypoactivity phenotype, along with increased anxiety-like behavior that is specific to the C57Bl/6J background.

The absence of memory deficits in the T29-1 cKO model was unexpected as deletion of ATRX in forebrain excitatory neurons results in hippocampal CA1 neuron alterations, including ultrastructural synaptic changes and reduced long-term potentiation (LTP), as well as impaired fear and spatial learning and memory [13,17] The observed differences between the T29-1 cKO and cKO^R1ag#5^ mice suggest that a more extensive loss of ATRX throughout the hippocampal formation and cortex is required to impair learning and memory. CA1 pyramidal excitatory neurons play a critical role in learning, memory consolidation, and retrieval, all of which depend on synaptic plasticity [42]. These neurons receive input either directly from the layer III entorhinal cortex (temporoammonic pathway), or indirectly through the layer II entorhinal cortex-dentate gyrus-hippocampal CA3 circuit (trisynaptic pathway) [42]. One explanation for the lack of cognitive deficits following T29-1 CaMKIIα-Cre-mediated loss of ATRX could be that functionally, the trisynaptic pathway remains intact, as some ATRX expression is retained in the entorhinal cortex, dentate gyrus and hippocampal CA3. Input from the trisynaptic pathway plays a critical role in learning and memory by facilitating NMDA receptor-dependent LTP at CA3-CA1 synapses [43–45]. This LTP is induced and regulated by a “band-pass filter” formed by the dentate gyrus and CA3 and allows input signals from the entorhinal cortex to propagate through the hippocampus [46]. Given that the dentate gyrus and CA3 both retain ATRX expression in our model, the lack of cognitive deficits could indicate that signal propagation in the trisynaptic pathway is unaffected by molecular changes caused by ATRX loss in hippocampal CA1. This is supported by a study showing that CA1-specifc loss of the NMDA-receptor 1 subunit, which inhibits signal propagation, results in spatial learning impairments [19]. Intact signal propagation could be the result of compensation, either within the ATRX-null CA1 neurons through alterations in intrinsic plasticity, or within the network activity, mediated by the function of interneurons [47]. It could also suggest that the molecular effects of ATRX loss in CA1 are restricted to specific layers, or strata. The trisynaptic and temporoammonic pathways target mid-apical and distal-apical CA1 dendrites, respectively, each expressing a unique composition of signal receptors [48]. Therefore, the molecular changes caused by ATRX loss in hippocampal CA1 may have a greater impact on one pathway than the other, such as the direct temporoammonic pathway which plays a role in memory consolidation [49]. Memory consolidation is the process by which recent memories, believed to be dependent on hippocampal activity, transition over time into remote memories, dependent on other brain structures, such as the anterior cingulate cortex [50–56]. Interestingly, T29-1 CaMKIIα-Cre-mediated loss of chromatin remodelling protein CTCF was shown to significantly impair remote cortex-dependent memory, while recent fear and spatial memory remained intact [55]. This suggests that T29-1 driven loss of chromatin remodeling proteins, such as CTCF or ATRX, may have a greater impact on remote cortical-dependent memory than on recent hippocampal-dependent memory. Unfortunately, the memory tests conducted on T29-1 CaMKIIα-Cre ATRX cKO mice represent recent long-term memory, which primarily relies on the hippocampus [55]. Future work should examine whether remote memory is altered in T29-1 cKO mice due to impaired signaling between the entorhinal cortex and hippocampal CA1 via the temporoammonic pathway.

There are two notable differences in T29-1 cKO mice compared to other ATRX models: spatial restriction of Cre-recombinase expression, previously discussed, and onset of expression at post-natal day 20 [25]. The lack of learning and memory deficits in T29-1 cKO mice could indicate that ATRX loss needs to occur earlier in hippocampal CA1 to have significant effects on cognition. Development of hippocampal CA1 begins at embryonic day 14 and continues postnatally until 3 months of age, with the majority of cells being produced between postnatal day 10–15, before ATRX loss in the T29-1 cKO mice [57]. The ability of the hippocampus to form memories occurs during post-natal day 20–24, and is dependent on circuit inhibition mediated by accumulation of extracellular perineuronal nets [58]. The formation of perineuronal nets is associated with the closure of critical periods, or a defined window of heightened plasticity required for circuit refinement and function [59–61]. Therefore, earlier loss of ATRX could affect critical processes such as neuronal development, connectivity, and network formation, while loss of ATRX at post-natal day 20 may be too late as it is occurring at the end of this critical period. The importance of timing of ATRX loss has been demonstrated previously when only an embryonic deletion of ATRX in forebrain excitatory neurons was sufficient to cause cognitive deficits and the presentation of autistic features [14,62]. Collectively, this suggests that a limitation of this model could be that loss of ATRX expression at later post-natal timepoints, during critical period closure, may not be sufficient to impair cognitive function.

We found that T29-1 CaMKIIα-Cre-mediated loss of ATRX results in strain-independent hypoactivity. This phenotype was also reported in knock-in mice harbouring the R246C patient mutation in the *Atrx* gene. Notably, these mice only display hypoactivity in late adulthood (1 year-old), while the hypoactivity observed in T29-1 cKO mice is present earlier (4−6 months-old) [37]. Additionally, reduced locomotor activity has been observed in multiple animal models with deletions or mutations of genes associated with intellectual disability, such as *Nlgn2*, *DLG3/SAP-102*, *Shank1*, and *Shank3* [63]. Reduced locomotor activity in the open field test could be interpreted as an attenuated response to novel situations, or attributed to poor performance in low-motivation situations [64,65]. However, poor mobility performance as a result of apathy is unlikely, as T29-1 cKO mice travel the same distance as their control counterparts in the EPM, which is also considered a low motivation environment [65]. Moreover, T29-1 cKO mice perform comparably to controls in swim speed and distance in the MWM, ruling out musculoskeletal deficits as an explanation for reduced activity. Further studies focusing on social and object novelty, as well as avoidance behaviours will help identify the underlying biological relevance of the observed hypoactivity.

Finally, by testing pure C57Bl/6J and hybrid C57Bl/6J + 129S2/Sv backgrounds, we were able to control for innate strain differences in behaviour [66–68]. We identified a strain-dependent increase in anxiety in the open field that was not replicated in the EPM. However, these tests measure two different aspects of anxiety: the anxious state versus the anxious trait [36,69]. Reduced time and distance in the center of the open field indicates that cKO^BL/6^ mice have an increased anxiety trait, which is a stable feature of the individual. Conversely, the normal preference for closed arms in the EPM suggests that the mice do not exhibit anxiety when confronted with an anxiogenic challenge, such as elevation and exposure to open, unprotected spaces [36,69]. Interestingly, the opposite phenotype is observed in the cKO^R1ag#5^ mice and other forebrain ATRX deletion models [13,14]. It has been demonstrated that increased hippocampal activity is linked to elevated anxiety providing an explanation for these correlated phenotypes in the cKO^BL/6^ mice [70]. Additionally, the observed strain-dependent effect on anxiety could be the result of innate strain differences. Compared to C57BL/6J, 129S2/Sv mice are naturally hypoactive and demonstrate elevated levels of anxiety during behavioural tests [66–68]. Therefore, it could be that an increase in anxiety determined based on movement would be more pronounced in the C57BL/6J background as they have a higher level of activity and a lower predisposition to anxiety. We found that the effect of T29-1 CaMKIIα-Cre-mediated loss of ATRX on spatial learning and memory was largely similar across both strain backgrounds suggesting that these cognitive phenotypes are not affected by genetic background.

## Conclusion

This work provides new information regarding the spatial and temporal contribution of ATRX function to hippocampal-dependent learning and memory. Our study also underscores the importance of ATRX function in pyramidal CA1 neurons for behavioural phenotypes such as hypoactivity and open-field exploration. Future studies should examine the impact of predominant CA1 ATRX loss on remote memory, novelty recognition, and avoidance behaviors, as well as its effects on signaling dynamics within hippocampal circuitry. Ultimately, this work demonstrates that disruption of ATRX across broader hippocampal and cortical regions is necessary to fully recapitulate the cognitive deficits associated with ATR-X syndrome.

## Supporting information

S1 Fig T29-1 CaMKIIα-Cre-mediated ATRX deletion in a hybrid background. (A) Representative immunofluorescence images of coronal brain sections from 3-month-old control and ATRX cKO hybrid C57Bl/6J + 129S2/Sv mice. Sections were stained for ATRX (red), the neuronal marker NeuN (green), and the nuclear marker DAPI (blue). Images were captured at 5X magnification; scale bar represents 500μm. Higher magnification images of ATRX and NeuN expression in (B) the hippocampal cornu ammonis 1 (CA1), (C) hippocampal CA2/3, (D) dentate gyrus (DG), and (E) the cortex (Ctx). Scale bars represent 250μm.
(TIF)

S2 Fig Validation of T29-1 CaMKIIα-Cre-mediated ATRX deletion in the ventral hippocampus. (A) Representative immunofluorescence images of the ventral hippocampus of the CA1 region from 3-month-old control and ATRX cKO C57Bl/6J and (B) hybrid mice. Sections were stained for ATRX (red), the neuronal marker NeuN (green), and the nuclear marker DAPI (blue). Scale bars indicate 1 mm.
(TIF)

S3 Fig Performance of R1ag#5 CaMKIIα-Cre ATRX cKO mice in open field, elevated plus maze, and contextual fear conditioning activity measures. (A) Comparison of total distance travelled between control mice in a pure C57Bl/6J and hybrid C57Bl/6J + 129S2/Sv strain background in the open field. (B) Time spent in the center of the open field apparatus for male control (n = 18) and control (n = 25) mice in a pure C57Bl/6J genetic background. (C) Time spent in the center of the open field male control (n = 17) and cKO (n = 13) mice in a hybrid genetic background. (D) Comparison of total distance travelled between control mice in a C57Bl/6J and hybrid genetic background in EPM. (E) Total distance traveled and (F) mean speed in the elevated plus maze (EPM) for male control (n = 18) and cKO (n = 25) mice in a pure C57Bl/6J genetic background. (G) total distance traveled and (H) mean speed in the EPM for male control (n = 17) and cKO (n = 13) mice in a hybrid genetic background. (I) Comparison of time spent immobile in 24-hour probe of contextual fear conditioning between control mice in a C57Bl/6J and hybrid genetic background in EPM. Statistical comparison across time was determined using two-way ANOVA; unpaired t-tests were used to compare total time in center, distance traveled, mean speed, and percent of time immobile. *p < 0.05, **** p < 0.00005.
(TIF)

S4 Fig Validation of R1ag#5 CaMKIIα-Cre ATRX knockout mice. Male B6.Cg-Tg(Camk2a-cre)2Szi/J mice were crossed with female mice harboring loxP-flanked (floxed) exon 18 of the *Atrx* gene to generate offspring on a hybrid C57BL/6J × 129S2/Sv background. Cre-mediated recombination in this model induces specific loss of ATRX in excitatory neurons of the forebrain structures beginning at postnatal day 5. Representative immunofluorescence images from coronal brain sections of 3-month-old male R1Ag#5-Camk2a-Cre mice illustrate ATRX deletion in (A) hippocampus and (B) caudal cortex. Sections were stained for ATRX (red), the neuronal marker NeuN (green), and the nuclear marker DAPI (blue). Scale bar: 200μm.
(TIF)

S5 Fig Performance of R1ag#5 CaMKIIα-Cre ATRX cKO mice in the Morris water maze. Spatial memory was assessed in probe trials following platform removal by measuring the percentage of time spent in each quadrant (A: Day 6 probe trials; B: Day 13 probe trials). Probe trials were analyzed using the Dirichlet uniformity test. Each colour column represents a sample, and each colour represents a quadrant. The hatched lines represent the mean fraction of time spent in each quadrant and the error bars on the means are approximated with the inverse Fisher information. In the Day 6 probe the fraction of time spent in the target quadrant is significantly higher, for both the Ctrl and cKO mice, leading to a non-uniform distribution (Ctrl: p = 0.00017; cKO p = 0.0078). In the Day 13 probe the fraction of time spent in the target quadrant is only significant for Ctrl mice (p = 0.0000066), whereas for cKO mice the fraction of time spent in each quadrant is approximately similar leading to a uniform distribution (p = 0.062). **p < 0.005, *** p < 0.0005.
(TIF)

S1 Table List of genotyping primers. List of primers used to validate mouse genotypes.
(DOCX)

S2 Table Summary of statistical analysis of behaviour experiments. Summary of statistics from all behaviour experiments conducted.
(DOCX)

## References

[1] Gibbons RJ, Picketts DJ, Villard L, Higgs DR. Mutations in a putative global transcriptional regulator cause X-linked mental retardation with alpha-thalassemia (ATR-X syndrome). Cell. 1995;80(6). 10.1016/0092-8674(95)90287-2 7697714

[2] Tissue expression of ATRX - Summary - The Human Protein Atlas. [cited 2026 Jan 13]. https://www.proteinatlas.org/ENSG00000085224-ATRX/tissue

[3] ATRX chromatin remodeler. NCBI Gene. https://www.ncbi.nlm.nih.gov/gene/546#gene-expression. Accessed 2026 Jan 12.

[4] Wong LH, McGhie JD, Sim M, Anderson MA, Ahn S, Hannan RD, et al. ATRX interacts with H3.3 in maintaining telomere structural integrity in pluripotent embryonic stem cells. Genome Res. 2010;20(3):351–60. doi: 10.1101/gr.101477.109 20110566 PMC2840985

[5] Voon HPJ, Hughes JR, Rode C, De La Rosa-Velázquez IA, Jenuwein T, Feil R, et al. ATRX Plays a Key Role in Maintaining Silencing at Interstitial Heterochromatic Loci and Imprinted Genes. Cell Rep. 2015;11(3):405–18. doi: 10.1016/j.celrep.2015.03.036 25865896 PMC4410944

[6] Watson LA, Solomon LA, Li JR, Jiang Y, Edwards M, Shin-ya K, et al. Atrx deficiency induces telomere dysfunction, endocrine defects, and reduced life span. J Clin Invest. 2013;123(5):2049–63. doi: 10.1172/JCI65634 23563309 PMC3635723

[7] Seah C, Levy MA, Jiang Y, Mokhtarzada S, Higgs DR, Gibbons RJ. Neuronal death resulting from targeted disruption of the Snf2 protein ATRX is mediated by p53. J Neurosci. 2008;28(47):12570–80. 19020049 10.1523/JNEUROSCI.4048-08.2008 PMC6671724

[8] Ritchie K, Seah C, Moulin J, Isaac C, Dick F, Bérubé NG. Loss of ATRX leads to chromosome cohesion and congression defects. J Cell Biol. 2008;180(2):315–24. doi: 10.1083/jcb.200706083 18227278 PMC2213576

[9] Levy MA, Kernohan KD, Jiang Y, Bérubé NG. ATRX promotes gene expression by facilitating transcriptional elongation through guanine-rich coding regions. Hum Mol Genet. 2015;24(7):1824–35. doi: 10.1093/hmg/ddu596 25452430

[10] Lewis PW, Elsaesser SJ, Noh K-M, Stadler SC, Allis CD. Daxx is an H3.3-specific histone chaperone and cooperates with ATRX in replication-independent chromatin assembly at telomeres. Proc Natl Acad Sci U S A. 2010;107(32):14075–80. doi: 10.1073/pnas.1008850107 20651253 PMC2922592

[11] Bérubé NG, Mangelsdorf M, Jagla M, Vanderluit J, Garrick D, Gibbons RJ, et al. The chromatin-remodeling protein ATRX is critical for neuronal survival during corticogenesis. J Clin Invest. 2005;115(2):258–67. doi: 10.1172/JCI22329 15668733 PMC544602

[12] Garrick D, Sharpe JA, Arkell R, Dobbie L, Smith AJH, Wood WG, et al. Loss of Atrx affects trophoblast development and the pattern of X-inactivation in extraembryonic tissues. PLoS Genet. 2006;2(4):e58. doi: 10.1371/journal.pgen.0020058 16628246 PMC1440874

[13] Tamming RJ, Dumeaux V, Jiang Y, Shafiq S, Langlois L, Ellegood J, et al. Atrx Deletion in Neurons Leads to Sexually Dimorphic Dysregulation of miR-137 and Spatial Learning and Memory Deficits. Cell Rep. 2020;31(13):107838. doi: 10.1016/j.celrep.2020.107838 32610139 PMC7326465

[14] Quesnel KM, Martin-Kenny N, Bérubé NG. A mouse model of ATRX deficiency with cognitive deficits and autistic traits. J Neurodev Disord. 2023;15(1):39. doi: 10.1186/s11689-023-09508-7 37957569 PMC10644498

[15] Ritchie K, Watson LA, Davidson B, Jiang Y, Bérubé NG. ATRX is required for maintenance of the neuroprogenitor cell pool in the embryonic mouse brain. Biol Open. 2014;3(12):1158–63. doi: 10.1242/bio.20148730 25395668 PMC4265753

[16] Tamming RJ, Siu JR, Jiang Y, Prado MAM, Beier F, Bérubé NG. Mosaic expression of Atrx in the mouse central nervous system causes memory deficits. Dis Model Mech. 2017;10(2):119–26. doi: 10.1242/dmm.027482 28093507 PMC5312007

[17] Gugustea R, Tamming RJ, Martin-Kenny N, Bérubé NG, Leung LS. Inactivation of ATRX in forebrain excitatory neurons affects hippocampal synaptic plasticity. Hippocampus. 2020;30(6):565–81. doi: 10.1002/hipo.23174 31713968

[18] Hunsaker MR, Kesner RP. Evaluating the differential roles of the dorsal dentate gyrus, dorsal CA3, and dorsal CA1 during a temporal ordering for spatial locations task. Hippocampus. 2008;18(9):955–64. doi: 10.1002/hipo.20455 18493930 PMC2570230

[19] Tsien JZ, Huerta PT, Tonegawa S. The essential role of hippocampal CA1 NMDA receptor-dependent synaptic plasticity in spatial memory. Cell. 1996;87(7):1327–38. doi: 10.1016/s0092-8674(00)81827-9 8980238

[20] Mueller SG, Chao LL, Berman B, Weiner MW. Evidence for functional specialization of hippocampal subfields detected by MR subfield volumetry on high resolution images at 4 T. Neuroimage. 2011;56(3). 10.1016/j.neuroimage.2011.03.028 PMC3085574 21419225

[21] Gerlai R. Hippocampal LTP and memory in mouse strains: Is there evidence for a causal relationship?. Hippocampus. 2002;12(5):657–66. doi: 10.1002/hipo.10101 12440580

[22] Nguyen PV, Abel T, Kandel ER, Bourtchouladze R. Strain-dependent differences in LTP and hippocampus-dependent memory in inbred mice. Learn Mem. 2000;7(3):170–9. doi: 10.1101/lm.7.3.170 10837506 PMC311331

[23] Montkowski A, Poettig M, Mederer A, Holsboer F. Behavioural performance in three substrains of mouse strain 129. Brain Res. 1997;762(1–2):12–8. doi: 10.1016/s0006-8993(97)00370-3 9262153

[24] Camp M, Norcross M, Whittle N, Feyder M, D’Hanis W, Yilmazer-Hanke D, et al. Impaired Pavlovian fear extinction is a common phenotype across genetic lineages of the 129 inbred mouse strain. Genes Brain Behav. 2009;8(8):744–52. doi: 10.1111/j.1601-183X.2009.00519.x 19674120 PMC2783364

[25] Tsien JZ, Chen DF, Gerber D, Tom C, Mercer EH, Anderson DJ, et al. Subregion- and cell type-restricted gene knockout in mouse brain. Cell. 1996;87(7):1317–26. doi: 10.1016/s0092-8674(00)81826-7 8980237

[26] Dragatsis I, Zeitlin S. CaMKIIalpha-Cre transgene expression and recombination patterns in the mouse brain. Genesis. 2000;26(2):133–5. doi: 10.1002/(sici)1526-968x(200002)26:2<133::aid-gene10>3.0.co;2-v 10686608

[27] Vorhees CV, Williams MT. Morris water maze: procedures for assessing spatial and related forms of learning and memory. Nat Protoc. 2006;1(2):848–58. doi: 10.1038/nprot.2006.116 17406317 PMC2895266

[28] Maugard M, Doux C, Bonvento G. A new statistical method to analyze Morris Water Maze data using Dirichlet distribution. F1000Res. 2019;8:1601. 31723422 10.12688/f1000research.20072.1 PMC6833991

[29] Mayford M, Wang J, Kandel ER, O’Dell TJ. CaMKII regulates the frequency-response function of hippocampal synapses for the production of both LTD and LTP. Cell. 1995;81(6):891–904. doi: 10.1016/0092-8674(95)90009-8 7781066

[30] Navarrete-Mathews M, Lee GS, Walerio A, Horne D, Wu Y, Zhou Y. Behavioral analyses of a forebrain glutamatergic neuron specific Ywhae conditional knockout mouse model. PLoS One. 2025;20(11):e0335427. doi: 10.1371/journal.pone.0335427 41218078 PMC12604760

[31] Matsuura K, Mohamed HMA, Youssef MMM, Yoshida Y, Yamamoto T. Synaptotagmin 2 is ectopically overexpressed in excitatory presynapses of a widely used CaMKΙΙα-Cre mouse line. iScience. 2022;25(8):104692. doi: 10.1016/j.isci.2022.104692 35856033 PMC9287804

[32] Sonner JM, Cascio M, Xing Y, Fanselow MS, Kralic JE, Morrow AL, et al. Alpha 1 subunit-containing GABA type A receptors in forebrain contribute to the effect of inhaled anesthetics on conditioned fear. Mol Pharmacol. 2005;68(1):61–8. doi: 10.1124/mol.104.009936 15833735

[33] Tatem KS, Quinn JL, Phadke A, Yu Q, Gordish-Dressman H, Nagaraju K. Behavioral and locomotor measurements using an open field activity monitoring system for skeletal muscle diseases. J Vis Exp. 2014;91:51785. 10.3791/51785 PMC4672952 25286313

[34] Seibenhener ML, Wooten MC. Use of the open field maze to measure locomotor and anxiety-like behavior in mice. J Vis Exp. 2015;96:e52434. 10.3791/52434 PMC4354627 25742564

[35] Ramos A. Animal models of anxiety: Do I need multiple tests?. Trends Pharmacol Sci. 2008;29(10):493–8. 18755516 10.1016/j.tips.2008.07.005

[36] Figueiredo Cerqueira MM, Castro MML, Vieira AA, Kurosawa JAA, Amaral Junior FL, Siqueira Mendes CC, et al. Comparative analysis between Open Field and Elevated Plus Maze tests as a method for evaluating anxiety-like behavior in mice. Heliyon. 2023;9(4):e14522. doi: 10.1016/j.heliyon.2023.e14522 37025809 PMC10070366

[37] Tillotson R, Yan K, Ruston J, DeYoung T, Córdova A, Turcotte-Cardin V, et al. A new mouse model of ATR-X syndrome carrying a common patient mutation exhibits neurological and morphological defects. Hum Mol Genet. 2023;32(15):2485–501. doi: 10.1093/hmg/ddad075 37171606 PMC10360390

[38] Nogami T, Beppu H, Tokoro T, Moriguchi S, Shioda N, Fukunaga K, et al. Reduced expression of the ATRX gene, a chromatin-remodeling factor, causes hippocampal dysfunction in mice. Hippocampus. 2011;21(6):678–87. doi: 10.1002/hipo.20782 20865721

[39] Anagnostaras SG, Gale GD, Fanselow MS. Hippocampus and contextual fear conditioning: Recent controversies and advances. Hippocampus. 2001;11(1):8–17. doi: 10.1002/1098-1063(2001)11:1<8::AID-HIPO1015>3.0.CO;2-7 11261775

[40] Maren S, Holt W. The hippocampus and contextual memory retrieval in Pavlovian conditioning. Behav Brain Res. 2000;110(1–2):97–108. doi: 10.1016/s0166-4328(99)00188-6 10802307

[41] Chu A, Gordon NT, DuBois AM, Michel CB, Hanrahan KE, Williams DC. A fear conditioned cue orchestrates a suite of behaviors in rats. eLife. 2024;13. 10.7554/eLife.82497 PMC11219038 38770736

[42] Basu J, Siegelbaum SA. The corticohippocampal circuit, synaptic plasticity, and memory. Cold Spring Harb Perspect Biol. 2015;7(11):a021733. doi: 10.1101/cshperspect.a021733 26525152 PMC4632668

[43] Stepan J, Dine J, Fenzl T, Polta SA, von Wolff G, Wotjak CT, et al. Entorhinal theta-frequency input to the dentate gyrus trisynaptically evokes hippocampal CA1 LTP. Front Neural Circuits. 2012;6:64. doi: 10.3389/fncir.2012.00064 22988432 PMC3439738

[44] Neunuebel JP, Knierim JJ. CA3 retrieves coherent representations from degraded input: direct evidence for CA3 pattern completion and dentate gyrus pattern separation. Neuron. 2014;81(2):416–27. 24462102 10.1016/j.neuron.2013.11.017 PMC3904133

[45] Barrientos SA, Tiznado V. Hippocampal CA1 subregion as a context decoder. J Neurosci. 2016;36(25):6602–4. 27335394 10.1523/JNEUROSCI.1107-16.2016 PMC6601752

[46] Stepan J, Dine J, Eder M. Functional optical probing of the hippocampal trisynaptic circuit in vitro: network dynamics, filter properties, and polysynaptic induction of CA1 LTP. Front Neurosci. 2015;9:160. doi: 10.3389/fnins.2015.00160 25999809 PMC4422028

[47] Sánchez-Aguilera A, Sánchez-Alonso JL, Vicente-Torres MA, Colino A. A novel short-term plasticity of intrinsic excitability in the hippocampal CA1 pyramidal cells. J Physiol (Lond). 2014;592(13):2845–64. 24756640 10.1113/jphysiol.2014.273185 PMC4221824

[48] Aksoy-Aksel A, Manahan-Vaughan D. The temporoammonic input to the hippocampal CA1 region displays distinctly different synaptic plasticity compared to the Schaffer collateral input in vivo: Significance for synaptic information processing. Front Synaptic Neurosci. 2013;5:5. doi: 10.3389/fnsyn.2013.00005 23986697 PMC3750210

[49] Remondes M, Schuman EM. Role for a cortical input to hippocampal area CA1 in the consolidation of a long-term memory. Nature. 2004;431(7009):699–703. doi: 10.1038/nature02965 15470431

[50] Frankland PW, Bontempi B, Talton LE, Kaczmarek L, Silva AJ. The involvement of the anterior cingulate cortex in remote contextual fear memory. Science. 2004;304(5672):881–3. 15131309 10.1126/science.1094804

[51] Teixeira CM, Pomedli SR, Maei HR, Kee N, Frankland PW. Involvement of the anterior cingulate cortex in the expression of remote spatial memory. J Neurosci. 2006;26(29):7555–64. 16855083 10.1523/JNEUROSCI.1068-06.2006 PMC6674278

[52] Goshen I, Brodsky M, Prakash R, Wallace J, Gradinaru V, Ramakrishnan C. Dynamics of retrieval strategies for remote memories. Cell. 2011;147(3):678–89. 22019004 10.1016/j.cell.2011.09.033

[53] Einarsson EÖ, Pors J, Nader K. Systems reconsolidation reveals a selective role for the anterior cingulate cortex in generalized contextual fear memory expression. Neuropsychopharmacology. 2015;40(2):480–7. doi: 10.1038/npp.2014.197 25091528 PMC4443963

[54] Bian XL, Qin C, Cai CY, Zhou Y, Tao Y, Lin YH. Anterior cingulate cortex to ventral hippocampus circuit mediates contextual fear generalization. J Neurosci. 2019;39(29):5728–39. 31097621 10.1523/JNEUROSCI.2739-18.2019 PMC6636085

[55] Kim S, Yu N-K, Shim K-W, Kim JI, Kim H, Han DH, et al. Remote memory and cortical synaptic plasticity require neuronal CCCTC-binding factor (CTCF). J Neurosci. 2018;38(22):5042–52. 29712785 10.1523/JNEUROSCI.2738-17.2018 PMC6705941

[56] Izquierdo I, Bevilaqua LRM, Rossato JI, Bonini JS, Medina JH, Cammarota M. Different molecular cascades in different sites of the brain control memory consolidation. Trends Neurosci. 2006;29(9):496–505. doi: 10.1016/j.tins.2006.07.005 16872686

[57] Fan S-J, Sun A-B, Liu L. Epigenetic modulation during hippocampal development. Biomed Rep. 2018;9(6):463–73. doi: 10.3892/br.2018.1160 30546873 PMC6256110

[58] Ramsaran AI, Wang Y, Golbabaei A, Aleshin S, de Snoo ML, Yeung BRA. A shift in the mechanisms controlling hippocampal engram formation during brain maturation. Science. 2023;380(6644):543–51. 37141366 10.1126/science.ade6530

[59] Wang D, Fawcett J. The perineuronal net and the control of CNS plasticity. Cell Tissue Res. 2012;349(1):147–60. doi: 10.1007/s00441-012-1375-y 22437874

[60] Hensch TK. Critical period plasticity in local cortical circuits. Nat Rev Neurosci. 2005;6(11):877–88. doi: 10.1038/nrn1787 16261181

[61] Ramsaran AI, Ventura S, Gallucci J, De Snoo ML, Josselyn SA, Frankland PW. A sensitive period for the development of episodic-like memory in mice. Curr Biol. 2025;35(9):2032-2048.e3. doi: 10.1016/j.cub.2025.03.032 40215964 PMC12055481

[62] Martin-Kenny N, Bérubé NG. Effects of a postnatal Atrx conditional knockout in neurons on autism-like behaviours in male and female mice. J Neurodev Disord. 2020;12(1):17. doi: 10.1186/s11689-020-09319-0 32580781 PMC7315487

[63] Luo J, Norris RH, Gordon SL, Nithianantharajah J. Neurodevelopmental synaptopathies: Insights from behaviour in rodent models of synapse gene mutations. Prog Neuropsychopharmacol Biol Psychiatry. 2018;84(Pt B):424–39. doi: 10.1016/j.pnpbp.2017.12.001 29217145

[64] Kouser M, Speed HE, Dewey CM, Reimers JM, Widman AJ, Gupta N. Loss of predominant Shank3 isoforms results in hippocampus-dependent impairments in behavior and synaptic transmission. J Neurosci. 2013;33(47):18448–68. 24259569 10.1523/JNEUROSCI.3017-13.2013 PMC3834052

[65] Cuthbert PC, Stanford LE, Coba MP, Ainge JA, Fink AE, Opazo P. Synapse-associated protein 102/dlgh3 couples the NMDA receptor to specific plasticity pathways and learning strategies. J Neurosci. 2007;27(10):2673–82. 17344405 10.1523/JNEUROSCI.4457-06.2007 PMC2851144

[66] Contet C, Rawlins JN, Deacon RM. A comparison of 129S2/SvHsd and C57BL/6JOlaHsd mice on a test battery assessing sensorimotor, affective and cognitive behaviours: implications for the study of genetically modified mice. Behav Brain Res. 2001;124(1):33–46. doi: 10.1016/s0166-4328(01)00231-5 11423164

[67] Abramov U, Puussaar T, Raud S, Kurrikoff K, Vasar E. Behavioural differences between C57BL/6 and 129S6/SvEv strains are reinforced by environmental enrichment. Neurosci Lett. 2008;443(3):223–7. doi: 10.1016/j.neulet.2008.07.075 18687379

[68] Võikar V, Vasar E, Rauvala H. Behavioral alterations induced by repeated testing in C57BL/6J and 129S2/Sv mice: implications for phenotyping screens. Genes Brain Behav. 2004;3(1):27–38. doi: 10.1046/j.1601-183x.2003.0044.x 14960013

[69] Goes TC, Almeida Souza TH, Marchioro M, Teixeira-Silva F. Excitotoxic lesion of the medial prefrontal cortex in Wistar rats: Effects on trait and state anxiety. Brain Res Bull. 2018;142:313–9. doi: 10.1016/j.brainresbull.2018.08.009 30120930

[70] Ghasemi M, Navidhamidi M, Rezaei F, Azizikia A, Mehranfard N. Anxiety and hippocampal neuronal activity: Relationship and potential mechanisms. Cogn Affect Behav Neurosci. 2022;22(3):431–49. doi: 10.3758/s13415-021-00973-y 34873665

