# Supplementary material for: ATRX function beyond hippocampal CA1 is required for cognitive deficits in mouse models of intellectual disability
Source: PLoS One. 2026 Apr 28;21(4):e0347770. doi: 10.1371/journal.pone.0347770 (PMC13123962; doi:10.1371/journal.pone.0347770)
Supplement: S1 Table — List of primers used to validate mouse genotypes. (DOCX) [file pone.0347770.s006.docx]

**S1 Table. List of Genotyping Primers.**

| **Allele** | **Forward Primer** | **Reverse Primer** | **Amplicon** |
| --- | --- | --- | --- |
| *Atrx* WT | AGA ACC GTT AGT GCA GGT TCA | TGA ACC TGG GGA CTT CTT TG | 988bp |
| *Atrx*^loxP^ | AGA ACC GTT AGT GCA GGT TCA | CCA CCA TGA TAT TCG GCA AG | ~1.2kb |
| αCaMKII-Cre | TGA CCA GAG TCA TCC TTA GCG | AAT GCT TCT GTC CGT TTG CC | ~750bp |

**S1 Table. List of Genotyping Primers**. List of primers used to validate mouse genotypes.
