# Supplementary material for: ATRX function beyond hippocampal CA1 is required for cognitive deficits in mouse models of intellectual disability
Source: PLoS One. 2026 Apr 28;21(4):e0347770. doi: 10.1371/journal.pone.0347770 (PMC13123962; doi:10.1371/journal.pone.0347770)
Supplement: S2 Table — Summary of statistics from all behaviour experiments conducted. (DOCX) [file pone.0347770.s007.docx]

**S2 Table. Summary of statistical analysis of behaviour experiments.** Summary of statistics from all behaviour experiments conducted.

| **Figure** | **Test** | **Parameter** | **Analysis** | **Results** | **Post-hoc Analysis** |
| --- | --- | --- | --- | --- | --- |
| 3A | OF | Distance Travelled over Time | 2-way ANOVA | main effect of time: F (23, 943) = 25.91, P<0.0001; main effect of genotype: F (1, 41) = 16.23, P=0.0002; genotype X time: F (23, 943) = 1.140, P=0.2936 |  |
| 3A | OF | Total Distance | Unpaired t test | t=4.029, df=41, F (17, 24) = 1.180, p=0.0002 |  |
| 3B | OF | Distance Travelled over Time | 2-way ANOVA | main effect of time: F (9.477, 255.9) = 31.83, P<0.0001; main effect of genotype: F (1, 27) = 4.882, P=0.0358; genotype X time: F (23, 621) = 1.039, P=0.4123 |  |
| 3B | OF | Total Distance | Unpaired t test with Welch's correction | t=2.390, df=20.33, F (15,12) = 6.471, p= 0.0266 |  |
| 4A | OF | Distance Travelled in Center over Time | 2-way ANOVA | main effect of time: F (6.931, 284.2) = 35.76, P<0.0001; main effect of genotype: F (1, 41) = 16.61, P=0.0002; genotype X time: F (23, 943) = 1.189, P=0.2456 |  |
| 4A | OF | Total Distance Travelled in Center | Unpaired t test | t=4.076, df=41, F (17, 24) = 1.188, p=0.0002 |  |
| 4A | OF | %(Distance Center/Total Distance Travelled) | Unpaired t test | t=2.163, df=41, F (17,24) = 2.179, p=0.0364 |  |
| 4B | EPM | % Time in Closed Arm | Unpaired t test | t=0.7895, df=41, F (17, 24) =1.580, p=0.4344 |  |
| 4B | EPM | % Time in Open Arm | Unpaired t test | t=0.5882, df=41, F (17, 24) =1.095, p=0.5596 |  |
| 4B | EPM | % Time in Center | Unpaired t test | t=0.7007, df=41, F (17, 24) =1.593, p=0.4875 |  |
| 4C | OF | Distance Travelled in Center over Time | 2-way ANOVA | main effect of time: F (7.680, 207.4) = 38.63, P<0.0001; main effect of genotype: F (1, 27) = 3.068, P=0.0912; genotype X time: F (23, 621) = 0.8163, P=0.7123 |  |
| 4C | OF | Total Distance Travelled in Center | Unpaired t test with Welch's correction | t=2.029, df=20.38, F (15, 11) = 6.808, p=0.0557 |  |
| 4C | OF | Total Distance Travelled in Center | Unpaired t test | t=0.6886, df=26, F (15, 11) = 1.316, p=0.4972 |  |
| 4D | EPM | % Time in Closed Arm | Unpaired t test | t=1.212, df=28, F (16, 12) = 1.044, p=0.2356 |  |
| 4D | EPM | % Time in Open Arm | Unpaired t test | t=0.4943, df=28, F (16, 12) = 1.012, p=0.6249 |  |
| 4D | EPM | % Time in Center | Unpaired t test | t=1.285, df=28, F (12, 16) = 1.316, p=0.2094 |  |
| 5A | CFC | Mean Speed over Time | 2-way ANOVA | main effect of time: F (5.786, 231.5) = 131.2, P<0.0001; main effect of genotype: F (1, 40) = 0.3014, P=0.5861; genotype X time: F (17, 680) = 2.548, P=0.0006 | Šídák's m.c. test |
| 5A | CFC | % Time Immobile over Time | 2-way ANOVA | main effect of time: F (9.877, 395.1) = 14.39, P<0.0001; main effect of genotype: F (1, 40) = 0.6916, P=0.4106; genotype X time: F (17, 680) = 0.5514, P=0.9264 |  |
| 5A | CFC | Pre-Shock vs Post-Shock Comparison | Multiple unpaired t tests | Ctrl: t=2.729, df=32, F (16, 16) = 1.306, p= 0.0102; cKO: t=3.588, df=48, F (24, 24) = 2.847, p= 0.0008 |  |
| 5B | CFC | Mean Speed over Time | 2-way ANOVA | main effect of time F (3.887, 104.9) = 120.2, P<0.0001; main effect of genotype: F (1, 27) = 0.3575, P=0.5549; genotype X time: F (17, 459) = 0.7551, P=0.7451 |  |
| 5B | CFC | % Time Immobile over Time | 2-way ANOVA | main effect of time: F (8.280, 223.5) = 12.40, P<0.0001; main effect of genotype: F (1, 27) = 0.2434, P=0.6257; genotype X time: F (17, 459) = 1.016, P=0.4392 |  |
| 5B | CFC | Pre-Shock vs Post-Shock Comparison | Multiple unpaired t tests | Ctrl: t=5.104, df=30, F (15, 15) = 1.290, p=<0.0001; cKO: t=3.680, df=24, F (12, 12) = 2.171, p=0.0012 |  |
| 5C | CFC | % Time Immobile over Time | 2-way ANOVA | main effect of time: F (7.495, 299.8) = 11.01, P<0.0001; main effect of genotype: F (1, 40) = 1.146, P=0.2909: genotype X time: F (11, 440) = 1.376, P=0.1811 |  |
| 5C | CFC | Total % Time Immobile | Unpaired t test | t=1.070, df=40, F (24, 16) = 1.253, p=0.2908 |  |
| 5D | CFC | % Time Immobile over Time | 2-way ANOVA | main effect of time: F (7.263, 196.1) = 4.972, P<0.0001; main effect of genotype: F (1, 27) = 3.909, P=0.0583; genotype X time: F (11, 297) = 0.5818, P=0.8434 |  |
| 5D | CFC | Total % Time Immobile | Unpaired t test | t=1.977, df=27, F (15, 12) = 2.601, p=0.0583 |  |
| 6A | MWM | Latency | 2-way ANOVA | main effect of day: F (2.445, 70.92) = 48.02, P<0.0001; main effect of genotype: F (1, 29) = 2.078, P=0.1601; genotype X day: F (3, 87) = 0.5676, P=0.6378 |  |
| 6B | MWM | Distance | 2-way ANOVA | main effect of day: F (2.558, 74.19) = 50.16, P<0.0001; main effect of genotype: F (1, 29) = 0.6386, P=0.4307; genotype X day: F (3, 87) = 0.7925, P=0.5013 |  |
| 6C | MWM | Speed | 2-way ANOVA | main effect of day: F (3, 87) = 6.036, P=0.0009; main effect of genotype: F (1, 29) = 2.364, P=0.1350; genotype X day: F (3, 87) = 1.723, P=0.1681 |  |
| 6D | MWM | 24-hour Probe Trial | 2-way ANOVA | main effect of quadrant: F (3, 116) = 42.62, P<0.0001; main effect of genotype: F (1, 116) = 3.618e-014, P>0.9999; interaction: F (3, 116) = 3.343, P=0.0217 | Dunnett’s m.c. test. CtrlBL/6: T vs. L p=0.0026, T vs. O p<0.0001, T vs. R p=0.0001; cKOBL/6: T vs. L p<0.0001, T vs. O p<0.0001, T vs. R p<0.0001 |
| 6E | MWM | 8-day Probe Trial | 2-way ANOVA | main effect of quadrant: F (3, 116) = 44.63, F (3, 116) = 44.63; main effect of genotype: F (1, 116) = 1.995e-014, P>0.9999; interaction: F (3, 116) = 7.312, P=0.0002 | Dunnett’s m.c. test. CtrlBL/6: T vs. L p=0.2261, T vs. O p<0.0001, T vs. R p=0.0091; cKOBL/6: T vs. L p<0.0001, T vs. O p<0.0001, T vs. R p<0.0001 |
| 6F | MWM | Latency | 2-way ANOVA | main effect of day: F (2.556, 71.56) = 133.1, P<0.0001; main effect of genotype: F (1, 28) = 5.965, P=0.0212; genotype X day: F (3, 84) = 1.545, P=0.2088 |  |
| 6G | MWM | Distance | 2-way ANOVA | main effect of day: F (2.701, 75.62) = 122.6, P<0.0001; main effect of genotype: F (1, 28) = 4.938, P=0.0345; genotype X day: F (3, 84) = 0.9072, P=0.4412 |  |
| 6H | MWM | Speed | 2-way ANOVA | main effect of day: F (2.466, 69.04) = 16.37, P<0.0001; main effect of genotype: F (1, 28) = 0.8503, P=0.3643; genotype X day: F (3, 84) = 0.5050, P=0.6799 |  |
| 6I | MWM | 24-hour Probe Trial | 2-way ANOVA | main effect of quadrant: F (3, 112) = 66.27, P<0.0001; main effect of genotype: F (1, 112) = 1.025e-013, P>0.9999; interaction: F (3, 112) = 2.384, P=0.0731 | Dunnett’s m.c. test. CtrlHybrid: T vs. L p <0.0001, T vs. O p<0.0001, T vs. R p <0.0001; cKOHybrid: T vs. L p<0.0001, T vs. O p<0.0001, T vs. R p<0.0001 |
| 6J | MWM | 8-day Probe Trial | 2-way ANOVA | main effect of quadrant: F (3, 112) = 71.05, P<0.0001; main effect of genotype: F (1, 112) = 6.571e-014, P>0.9999; interaction: F (3, 112) = 3.156, P=0.0276 | Dunnett’s m.c. test. CtrlHybrid: T vs. L p <0.0001, T vs. O p<0.0001, T vs. R p <0.0001; cKOHybrid: T vs. L p<0.0001, T vs. O p<0.0001, T vs. R p<0.0001 |
| 7A | OF | Distance Travelled over Time | 2-way ANOVA | main effect of time: F (2.351, 42.31) = 25.48, P<0.0001; main effect of genotype: F (1, 18) = 2.792, P=0.1121; genotype X time: F (23, 414) = 3.745, P<0.0001 | Šídák's m.c. test |
| 7A | OF | Total Distance | Unpaired t test | t=1.671, df=18, F (9, 9) = 1.346, p=0.1121 |  |
| 7B | OF | Time in Center over Time | 2-way ANOVA | main effect of time: F (3.596, 64.73) = 18.70, P<0.0001; main effect of genotype: F (1, 18) = 1.275, P=0.2737; genotype X time: F (23, 414) = 3.340, P<0.0001 | Šídák's m.c. test, 5min: p=0.0157 |
| 7B | OF | Total Time in Center | Unpaired t test | t=1.129, df=18, F (9, 9) = 3.385, p=0.2737 |  |
| 7C | OF | Distance Travelled in Center over Time | 2-way ANOVA | main effect of time: F (3.804, 68.47) = 33.11, P<0.0001; main effect of genotype: F (1, 18) = 1.743, P=0.2033; genotype X time: F (23, 414) = 2.178, P=0.0015 | Šídák's m.c. test |
| 7C | OF | Total Distance Travelled in Center | Unpaired t test | t=1.320, df=18, F (9, 9) = 1.324, p=0.2033 |  |
| 7D | EPM | % Time in Closed Arm | Unpaired t test | t=1.707, df=18, F (9, 9) = 1.124, p=0.105 |  |
| 7D | EPM | % Time in Open Arm | Unpaired t test with Welch's correction | t=2.924, df=11.08, F (9, 9) = 8.537, p=0.0138 |  |
| 7D | EPM | % Time in Center | Unpaired t test | t=0.3942, df=18, F (9, 9) = 3.734, p=0.6981 |  |
| 8A | CFC | Mean Speed over Time | 2-way ANOVA | main effect of time: F (4.569, 82.25) = 27.60, P<0.0001; main effect of genotype: F (1, 18) = 3.119, P=0.0943; genotype X time: F (17, 306) = 2.594, P=0.0006 | Šídák's m.c. test |
| 8A | CFC | % Time Immobile over Time | 2-way ANOVA | main effect of time: F (7.721, 139.0) = 5.783,  P<0.0001; main effect of genotype: F (1, 18) = 3.317, P=0.0852; genotype X time: F (17, 306) = 2.818, P=0.0002 | Šídák's m.c. test |
| 8A | CFC | Pre-Shock vs Post-Shock Comparison | Multiple unpaired t tests | Ctrl: t=2.189, df=18, F ( 9, 9) = 2.841, p=0.0420; cKO: t=3.819, df=18, F (9, 9) = 4.460, p=0.0013 |  |
| 8B | CFC | % Time Immobile over Time | 2-way ANOVA | main effect of time: F (6.022, 108.4) = 2.048, P=0.0651; main effect of genotype: F (1, 18) = 83.41, P<0.0001; genotype X time: F (11, 198) = 2.061, P=0.0248 | Šídák's m.c. test. 60sec: p=0.0026; 90sec: p=0.0068; 120sec: p <0.0001; 150sec: p <0.0001; 180sec: p <0.0001; 210sec: p=0.0006; 240sec: p=0.0003; 270sec: p=0.0088; 300sec: p=0.0027; 330sec: p=0.0001; 360sec: p=0.0006 |
| 8B | CFC | Total % Time Immobile | Unpaired t test | t=9.133, df=18, F (9, 9) = 1.088, p<0.0001 |  |
| 8C | MWM | Latency | 2-way ANOVA | main effect of day: F (3.053, 51.90) = 51.01, P<0.0001; main effect of genotype: F (1, 17) = 31.18, P<0.0001; genotype X day: F (4, 68) = 1.692, P=0.1620 |  |
| 8D | MWM | Distance | 2-way ANOVA | main effect of day: F (2.799, 47.58) = 43.67, P<0.0001; main effect of genotype: F (1, 17) = 30.37, P<0.0001; genotype X day: F (4, 68) = 1.012, P=0.4076 |  |
| 8E | MWM | Speed | 2-way ANOVA | main effect of day: F (3.046, 51.78) = 8.788, P<0.0001; main effect of genotype: F (1, 17) = 0.04412, P=0.8361; genotype X day: F (4, 68) = 0.1092, P=0.9789 |  |
| 8F | MWM | 24-hour Probe Trial | 2-way ANOVA | main effect of quadrant: F (3, 68) = 20.41, P<0.0001; main effect of genotype: F (1, 68) = 3.471e-014, P>0.9999; interaction: F (3, 68) = 1.068, P=0.3683 | Dunett’s m.c. test. CtrlR1ag#5: T vs. L p=0.0003, T vs. O p <0.0001, T vs. R p=0.0004; cKOR1ag#5: T vs. O p <0.0001, T vs. R p=0.0002 |
| 8G | MWM | 8-day Probe Trial | 2-way ANOVA | main effect of quadrant: F (3, 68) = 10.74, P<0.0001; main effect of genotype: F (1, 68) = 7.414e-014, P>0.9999; interaction: F (3, 68) = 3.980, P=0.0113 | Dunett’s m.c. test. CtrlR1ag#5: T vs. L p=0.0225, T vs. O p <0.0001, T vs. R p=0.0455; cKOR1ag#5: T vs. R p=0.0015 |
| Supplemental 3A | OF | Total Distance | Unpaired t test | t=8.671, df=32, F (17, 15) = 2.455, p<0.0001 |  |
| Supplemental 3B | OF | Time in Center over Time | 2-way ANOVA | main effect of time: F (7.291, 298.9) = 28.96, P<0.0001; main effect of genotype: F (1, 41) = 4.296, P=0.0445; genotype X time: F (23, 943) = 1.341, P=0.1305 |  |
| Supplemental 3B | OF | Total Time in Center | Unpaired t test | t=2.073, df=41, F (17, 24) = 1.186, p=0.0445 |  |
| Supplemental 3C | OF | Time in Center over Time | 2-way ANOVA | main effect of time: F (4.282, 115.6) = 37.38, P<0.0001; main effect of genotype: F (1, 27) = 2.361, P=0.1361; genotype X time: F (23, 621) = 0.6170, P=0.9190 |  |
| Supplemental 3C | OF | Total Time in Center | Unpaired t test with Welch's correction | t=1.001, df=13.55, F (11, 15) = 6.500, p=0.3341 |  |
| Supplemental 3D | EPM | Total Distance | Unpaired t test | t=1.447, df=41, F (17, 24) = 1.203, p=0.1555 |  |
| Supplemental 3E | EPM | Mean Speed | Unpaired t test | t=1.422, df=41, F (17, 24) = 1.212, p=0.1626 |  |
| Supplemental 3F | EPM | Total Distance | Unpaired t test | t=1.976, df=28, F (16, 12) = 2.478, p=0.058 |  |
| Supplemental 3G | EPM | Mean Speed | Unpaired t test | t=1.919, df=28, F (16, 12) = 2.418, p=0.0652 |  |
| Supplemental 3H | EPM | Total Distance | Unpaired t test | t=5.025, df=33, F (17, 16) = 2.697, p<0.0001 |  |
| Supplemental 3I | CFC | Total % Time Immobile | Unpaired t test | t=2.643, df=32, F (17, 15) = 1.660, p=0.0126 |  |

m.c. = multiple comparisons; OP = open field; EPM = elevated plus maze; CFC = contextual fear conditioning; MWM = Morris water maze

**S2 Table. Summary of statistical analysis of behaviour experiments.** Summary of statistics from all behaviour experiments conducted.
